# All-oral low-dose chemotherapy TEPIP is effective and well-tolerated in patients with peripheral T-cell lymphoma

**DOI:** 10.3389/fonc.2023.1177330

**Published:** 2023-05-26

**Authors:** Matthias A. Fante, Dennis C. Harrer, Barbara Zartner, Florian Lüke, Stephanie Mayer, Karin Menhart, Albrecht Reichle, Wolfgang Herr, Martin Vogelhuber, Daniel Heudobler

**Affiliations:** ^1^ Department of Internal Medicine III, Hematology and Internal Oncology, University Hospital Regensburg, Regensburg, Germany; ^2^ Division of Personalized Tumor Therapy, Fraunhofer Institute for Toxicology and Experimental Medicine, Regensburg, Germany; ^3^ Department of Nuclear Medicine, University Hospital Regensburg, Regensburg, Germany; ^4^ Bavarian Cancer Research Center (BZKF), University Hospital Regensburg, Regensburg, Germany

**Keywords:** TEPIP, relapsed/refractory PTCL, PTCL, metronomic chemotherapy, all-oral treatment, relapsed lymphoma, palliative treatment

## Abstract

**Purpose:**

Peripheral T-cell lymphoma (PTCL) is a rare and heterogenous hematologic malignancy with poor prognosis especially in elderly and frail patients who are not eligible for intensive treatment. The resulting palliative setting necessitates tolerable but effective schedules for outpatient treatment. TEPIP is a locally developed, all-oral low-dose regimen comprising trofosfamide, etoposide, procarbazine, idarubicin, and prednisolone.

**Methods:**

In this observational retrospective, single-center study, the safety and efficacy of TEPIP was evaluated in 12 patients (pts.) with PTCL treated at the University Medical Center Regensburg between 2010 and 2022. The endpoints were overall response rate (ORR) and overall survival (OS), and adverse events were individually reported according to the Common Terminology Criteria for Adverse Events (CTCAE) criteria.

**Results:**

The enrolled cohort was characterized by advanced age (median 70 years), extensive disease (100% Ann Arbor ≥stage 3), and poor prognosis (75% high/high-intermediate international prognostic index). The most common subtype was angioimmunoblastic T-cell lymphoma (8/12), and 11/12 patients had relapsed or refractory disease at TEPIP onset with a median of 1.5 prior treatment regimens. After a median of 2.5 TEPIP cycles (total of 83 cycles), the ORR was 42% (complete remission 25%), and the OS reached a median of 185 days. Any grade of adverse event (AE) occurred in 8/12 patients, with four patients showing AE ≥CTCAE grade 3 (33%), and the AEs were mainly non-hematological.

**Conclusion:**

TEPIP demonstrated competitive efficacy with a tolerable safety profile in a highly palliative cohort of patients with difficult-to-treat PTCL. The all-oral application, which makes outpatient treatment possible, is particularly noteworthy.

## Introduction

We recently reported promising safety and efficacy data of the locally developed all-oral, low-dose chemotherapy regimen TEPIP (trofosfamide, etoposide, procarbazine, idarubicin, prednisolone) in relapsed/refractory (R/R) high-grade B cell lymphoma contributing to quality of life by enabling outpatient treatment in a palliative setting (1). Peripheral T-cell lymphomas (PTCL) represent a further rare and heterogenous group of non-Hodgkin lymphoma (2) which is also lacking in effective treatment options in the palliation of patients (pt.) in a R/R state. PTCL with its most common subtypes PTCL not otherwise specified (PTCL-NOS), angioimmunoblastic T-cell lymphoma (AITL), and anaplastic large cell lymphoma (ALCL) (3) exhibit a more aggressive pathobiology as compared with B cell lymphoma showing a dismal overall survival (OS) (4–6).

The established first-line treatment consists of an anthracycline-based multi-agent chemotherapy CHOP backbone (cyclophosphamide, doxorubicin, vincristine, and prednisolone) followed by consolidative high-dose chemotherapy and autologous stem cell transplantation (ASCT) in responding and transplant-eligible patients if considered appropriate. In younger (<60 years) patients, CHOP might be complemented by etoposide to improve event-free survival and progression-free survival (PFS) (7, 8). A recent therapy adjustment was made under the impact of the phase III ECHELON-2 study, which demonstrated a significant improvement of overall response rate (ORR), PFS, and OS in combination with the anti-CD30 antibody brentuximab-vedotin (*i*.*e*., BV-CHP) and resulted in the approval for CD30^+^ PTCL (US Food and Drug Administration) and ALCL (European Medicines Agency (EMA)), respectively (9). Despite all efforts to optimize upfront therapy, the rates of primary refractory and relapsed patients with poor prognosis remain high at up to 70% (10–12), and salvage chemotherapy with consecutive allogeneic stem cell transplantation might be the only option for durable disease control in this high-risk constellation.

In frail and transplant-ineligible patients receiving dose-attenuated CHOP, the 2-year PFS (37%) and OS (47%) are disappointing (13), and the treatment of R/R PTCL in this vulnerable cohort remains challenging. A retrospective analysis from the COMPLETE registry has demonstrated the superiority of a single agent to combination chemotherapy in R/R PTCL (14). The FDA-approved [but not EMA-approved] single-agent therapy antifolate pralatrexate and the histone-deacetylase inhibitors romidepsin and belinostat have shown moderate ORR of approximately 30% and tolerable safety profiles (15–17). Furthermore, special attention is paid to recent studies investigating the efficacy of hypomethylating agents in PTCL with mutations in epigenetic regulators (*e*.*g*., TET2), which have yielded promising results (18, 19) and justified the initiation of further studies.

While conventional chemotherapies aim for a maximally tolerated dose, metronomic regimens combine low-dose agents to overcome therapy resistance and reduce toxicity while targeting both the tumor cells and the tumor-promoting microenvironment (20–23). To the best of our knowledge, only a few groups have focused on suchlike regimens in PTCL, however with partially encouraging results (24–27).

In aggregate, we would like to raise awareness for the all-oral, prolonged low-dose chemotherapy regimen TEPIP (trofosfamide, etoposide, procarbazine, idarubicin, prednisolone) as an effective therapeutic option in patients suffering from R/R PTCL with special emphasis on the use in outpatient setting.

## Methods

In this retrospective single-center study, we analyzed the efficacy and safety of the all-oral, low-dose chemotherapy TEPIP administered in 12 patients at the University Medical Center Regensburg (UKR) between 2010/01/01 and 2022/12/31. All patients were treated on a compassionate-use basis. We identified the cohort by an in-clinic, medical file database query using the term “TEPIP” and considered only T-cell lymphomas, excluding mixed histopathologies (*e*.*g*., NK/T-cell lymphoma). Due to the retrospectivity and the outpatient drug administration, clinical parameters and histologic diagnoses were obtained from medical reports, resulting in a partially limited data set. The analysis was approved by the local Ethics Committee (reference number: 23- 3250-104) and performed in compliance with the current Declaration of Helsinki. All cases analyzed were pseudonymized, and patients who were alive gave written informed consent for publication.

### Chemotherapy regimen

**Figure 1:**
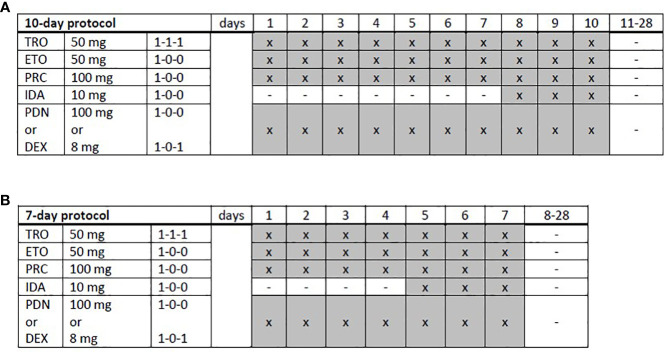
Trofosfamide, etoposide, procarbazine, idarubicin, and prednisolone schedules. **(A)** A full-dose, 10-day or **(B)** a dose-reduced, 7-day protocol was applied depending on the patient’s performance status and expected toxicity tolerance. TRO, trofosfamide; ETO, etoposide; PRC, procarbazine; IDA, idarubicin; PDN, prednisolone; DEX, dexamethasone; x, administration; hyphen, no administration.

TEPIP was administered as an all-oral chemotherapy regimen (**Figure 1**), allowing a fully outpatient treatment, and comprised of trophosphamide at 50 mg (1-1-1, abs. 150 mg), etoposide at 50 mg (1–0–0), procarbazine at 100 mg (1-0-0), and prednisolone at 100 mg (1-0-0) on days 1 to 10, which was shortened to 7 days in case of numerous pre-treatments (equal to or more than two lines of therapy) or advanced age (biological age ≥65 years) as necessary in the majority of our cohort. On days 8 to 10 and 5 to 7, respectively, a daily single dose at 10 mg was added. The course was repeated every 28 days provided that the leukocyte count exceeded 3,000/µl and continued until disease progression or the occurrence of adverse events. All patients treated with TEPIP received sulfamethoxazole-trimethoprim and acyclovir as prophylactic therapies for infections. Antifungal agents or additional antibiotics, such as quinolones, were just initiated in case of suspected infections. Apart from an appropriate antiemesis (*e*.*g*., metoclopramide), no specific supportive therapy (*e*.*g*., granulocyte-colony-stimulating factor) was administered.

### Disease classification and response assessment

At first diagnosis and TEPIP onset, lymphoma disease was staged according to the Ann Arbor criteria. All patients underwent bone marrow biopsy. Prognosis as assessed by the use of the International Prognostic Index (IPI) score is reported. Response assessment was conducted as clinically indicated without fixed schedules, as most patients were treated in an outpatient, palliative setting. Therefore, response was reported as the best response documented by reports of CT or PET-CT imaging according to the 2014 Lugano criteria (28). For patients treated in recent years, staging was performed with PET-CT imaging (patients 3, 4, 10, 11, and 12). Prior to 2020, CT imaging was the standard for response assessment.

### Adverse event assessment

Toxicities are listed as reported by outpatient reports and graded according to the Common Terminology Criteria for Adverse Events (CTCAE) version 5.0.

### Statistical analysis

Analyses were performed using PRISM 5 (GraphPad, San Diego, CA, USA) and SPSS 28 (IBM, Armonk, NY, USA). The ORR was defined as the sum of patients acquiring complete (CR) or partial remission (PR). Survival was analyzed as OS covering the period between the onset of TEPIP treatment and the patient’s death or the end of the observation period (2022/12/31), respectively. PFS was calculated as the difference between the onset of TEPIP treatment and the diagnosis of relapse or patient’s death, whereas duration of response (DOR) was defined as the period between a primarily recorded response and the subsequent progression of disease in responding patients. The endpoints OS and PFS (median and estimated OS/PFS at 6 and 12 months ± standard error) were depicted as Kaplan–Meier curves.

## Results

### Patient characteristics and response to TEPIP therapy

**Figure 2:**
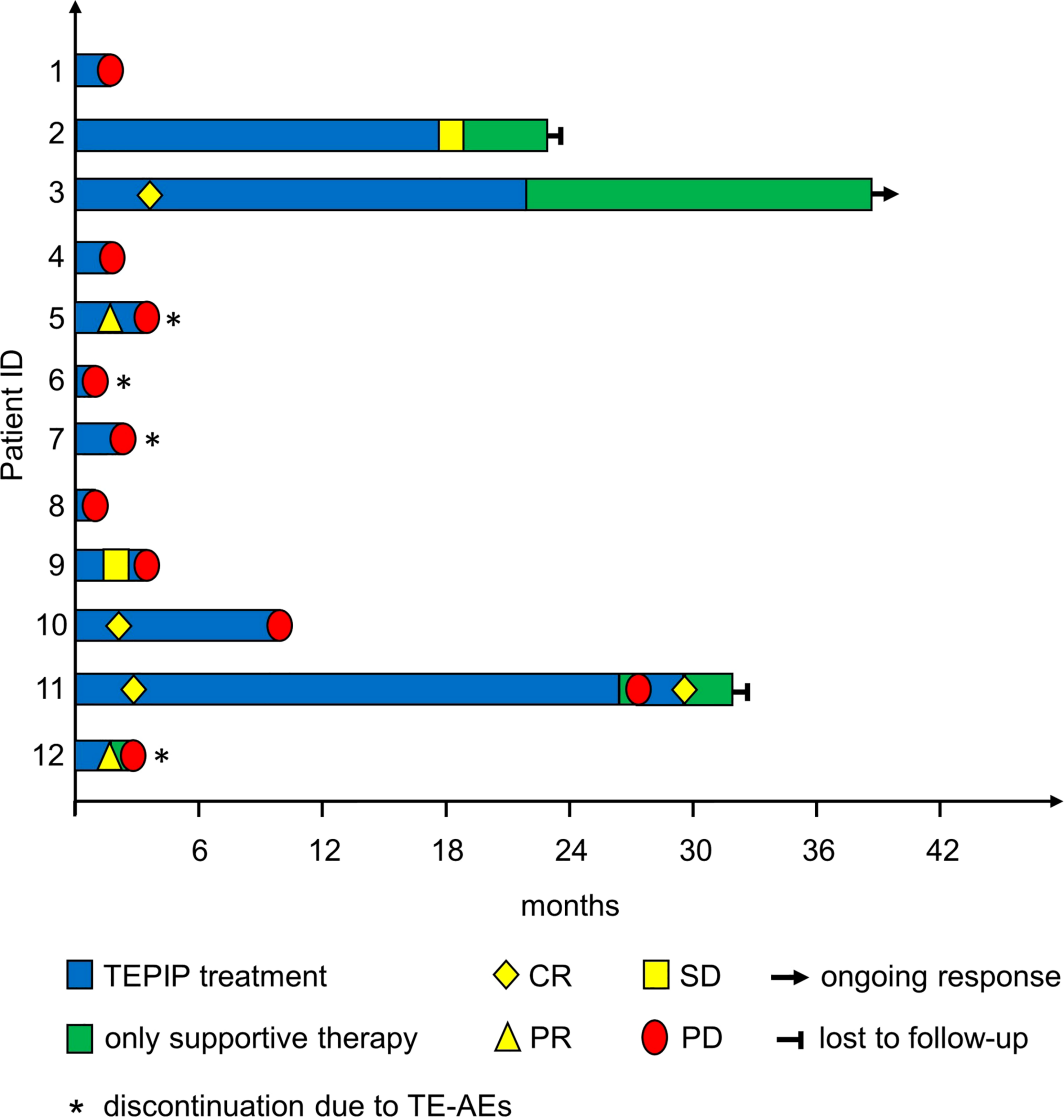
Treatment and response of the included patients during the treatment course as depicted by a swimmer plot.

**Figure 3:**
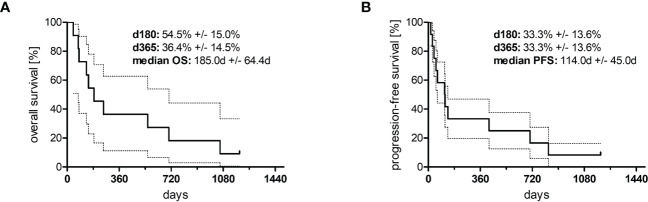
Kaplan–Meier curves of **(A)** overall survival (*n* = 10) and **(B)** progression-free survival (*n* = 11). The area between the dotted lines represents the standard error.

In total, 12 adult patients (five female and seven male) with histologically confirmed PTCL were treated at our medical center on a compassionate-use basis with the all-oral low-dose chemotherapy regimen TEPIP based on the daily application of low-dose trofosfamide, etoposide, procarbazine, idarubicin, and prednisolone (**Figure 1**). The baseline patient characteristics, including previous lines of therapy, are depicted in **Table 1**. The median age was 70 years (range, 43–84). AITL represented the most common T-cell lymphoma subtype (8 pt.). Prior to TEPIP initiation, all patients presented with stage 3 or higher according to the Ann Arbor classification accompanied by IPI scores predominantly at the high-intermediary level. A total of 11 patients were subjected to TEPIP treatment after having failed up to four different lines of therapy, including ASCT (1 pt.) and experimental checkpoint blockade with nivolumab (1 pt.). Only one patient did not receive any treatment before starting TEPIP. The clinical responses to TEPIP are documented in **Table 2**, and individual clinical courses are shown in **Figure 2**. Each patient underwent on average (median) of 2.5 cycles (range, 1 to 24) of TEPIP treatment, and a total of 83 cycles was applied. The median OS was 185 days (± 64.4 days, *n* = 11), and the median PFS amounted to 114 days (± 45.0 days, *n* = 12) (**Figure 3**). The overall response rate (CR + PR) was 42%, and the median duration of response was 10 months (range, 1–35 months). Three patients, including a heavily pretreated patient relapsing from high-dose chemotherapy and subsequent autologous stem cell transplantation (pt. 11), achieved complete remission (25%) in response to TEPIP treatment. At the time of this writing (February 2023), one patient (pt. 3) is still in complete remission despite the discontinuation of TEPIP treatment, while relapse was observed in the other complete responders after 10 months (patient 10) and 25 months (pt. 11), respectively. Remarkably, re-initiation of TEPIP treatment could re-induce complete remission in patient 11. Thereafter, TEPIP had to be permanently stopped due to pancytopenia originating from arising secondary acute myeloid leukemia, which necessitated switching to 5-azacitidine treatment. Moreover, two patients displayed transient partial remissions of their T-cell lymphoma lesions, and stable disease could be observed in two additional patients. Approximately half of all patients were primary refractory to TEPIP treatment. Those patients failing TEPIP treatment were either switched to best supportive care treatment or were continued on individual protocols as depicted in **Table 2**. In summary, the all-oral TEPIP treatment conducted in an outpatient setting harbors the potential for durable remissions in heavily pretreated patients with advanced T-cell lymphoma.

**Table 1:** Characteristics of T-cell lymphoma patients.

Patient ID	Sex	Age^a^	Subtype	Ann Arbor staging	IPI^a^	Pre-treatment
		(years)		at 1st dx/TEPIP start		
1	Male	70	AITL	IIA/IIIA	Low	6 × CHOP 4 × DHAC
2	Female	76	AITL	IIIB/IIIA	High-intermediate	6 × CHOP
3	Female	72	AITL	IIIA/IVA	Low-intermediate	12 × CHOP
4	Female	70	AITL	IIIA/IIIA	Low-intermediate	6 × CHOP
5	Male	45	AITL	IVA/IVA	High-intermediate	5 × R-CHOEP 1 × DHAP
6	Male	79	PTCL-NOS	IVAE/IVAE	High-intermediate	Prednisolone Vincristine 3 × CHOP
7	Male	84	AITL	-/IVA	High	None
8	Male	43	HSTL	IVB/IVB	High-intermediate	3 × CHOEP 1 × DHAP Fludarabine Alemtuzumab
9	Female	71	AITL	IVB/IIIA	High-intermediate	6 × CHOP 2 × DHAC 8 × BV
10	Female	67	FTCL	IVAE/IVAE	High-intermediate	6 × CHOEP 6 × N/Gem/Ox MTX weekly
11	Male	66	AITL	IIIA/IIIA	High-intermediate	5 × CHOP 1 × DHAP/1 × DHAC BEAM + ASCT 2 × Gem/Ox
12	Male	66	ALCL	IVA/IVA	High-intermediate	6 × BV-CHP

ATIL, angioimmunoblastic T-cell lymphoma; PTCL-NOS, peripheral T-cell lymphoma not otherwise specified; HSTL, hepatosplenic T-cell lymphoma; FTCL, follicular T-cell lymphoma; ALCL, anaplastic large cell lymphoma; CHOEP, cyclophosphamide, doxorubicin, vincristine, etoposide, and prednisolone; DHAP/C, dexamethasone, cytarabine, cisplatin/carboplatin; BV, brentuximab vedotin; N/Gem/Ox, nivolumab, gemcitabine, oxaliplatin; BEAM, BCNU, etoposide, cytarabine, and melphalan; ASCT, autologous stem cell transplantation; CHP, cyclophosphamide, doxorubicin, and prednisolone.

a At first diagnosis.

**Table 2:** Response to trofosfamide, etoposide, procarbazine, idarubicin, and prednisolone (TEPIP) therapy.

Patient ID	TEPIP cycles	Best response^a^	OS^b^	Subsequent therapy
	(n)	(DOR, months)^c^	(days)	
1	2	PD	77	BSC
2	16	SD (18)	704	BSC
3	15	CR (35+)	1193*	No treatment
4	2	PD	185	Gemcitabine/carboplatin/dexamethasone
5	2	PR (2)	82	BSC
6	1	PD	NA^d^	Cyclophosphamide/etoposid/procarbazine/prednisolone
7	3	PD	148	BSC
8	1	PD	42	BSC
9	4	SD (1)	134	Lomustin
10	11	CR (10)	558	Belinostat
11	24	CR (25)	218	5-Azacitidine
12	2	PR (1)	251	BSC
median	2.5	10 (DOR)	185	

DOR, duration of response; PD, progressive disease; SD, stable disease; PR, partial remission; CR, complete remission; BSC, best supportive care; NA, not available.

a Response to TEPIP therapy.

b Overall survival of patients, (the symbol “*” signifies being still alive).

c Duration of response in the case of SD/PR/CR (the symbol “+” signifies an ongoing response).

d Overall survival not available because of loss to follow-up.

### Analysis of the versatile therapeutic potential of TEPIP in patients with PTCL based on five clinical courses

Out of the 12 patients with PTCL treated at our center, the clinical courses of five patients (patient ID numbers 3, 5, 10, 11, and 12 according to **Tables 1** and **2**) particularly emphasize the versatile therapeutic potential of TEPIP in PTCL patients.

#### Case 1 (pt. 3)

A 72-year-old female patient presented with progressive localized axillary lymphadenopathy to our department. After a diagnosis of AITL, treatment with 6x CHOP-14 helped her achieve complete remission, but it had to be repeated due to relapse at 27 months later. After a further six cycles, a long-lasting (6.5 years) CR was observed. Thereafter, the patient suffered again from stage IVA [disseminated lymph nodes (LN), 1% to 2% bone marrow (BM) infiltration] relapse of the known AITL, which was molecularly characterized as ALK1-negative, TP53/17p del-negative, and TET2-positive. Due to the advanced patient age, a palliative treatment with all-oral TEPIP without idarubicin (q4w, d1-7) was initiated. After 4 months, a CR was observed despite dose reductions (trofosfamide, prednisolone, and procarbazine) and a stretched cycle duration from 4 to 6 weeks due to limited tolerability (fatigue). TEPIP was discontinued after 23 months due to multiple cutaneous squamous cell carcinomas with persistent complete remission. Until the end of the observational period (31-Dec-2022), no relapse has occurred.

Conclusion: Patient case 1 demonstrates a durable response to TEPIP treatment in an elderly and frail patient, which allowed dose adjustment to achieve individual treatment tolerability.

#### Case 2 (pt. 5)

A 45-year-old male patient presented with a liver-infiltrating stage IVB AITL (additional BM infiltration) and simultaneous EBV-positive DLBCL. During the first-line treatment with 5x R-CHOEP, the patient experienced several severe adverse events, and a CT scan and bone marrow puncture before the planned sixth course showed a massive progression of AITL. However, no persisting evidence of DLBCL was found. Therapy was rotated to the salvage regimen DHAP in the intention of later high-dose (HD) chemotherapy and ASCT. A pneumogenic sepsis interrupted the treatment course, resulting in another rapid progression. As a consequence, TEPIP treatment (without idarubicin) was initiated, which achieved prompt and impressive PR (resolved liver and minimal pulmonary manifestation). After a second course, the patient developed pancytopenia that necessitated a protracted recovery period, which led to progression and death due to failure of lymphoma-infiltrated organs.

Conclusion: Patient case 2 identifies TEPIP as a treatment option in refractory AITL, however, at the expense of potentially serious adverse events such as life-threatening cytopenia.

#### Case 3 (pt. 10)

A 67-year-old female patient was referred to the Department of Dermatology at the University Hospital Regensburg with pruritus, eczema, and subcutaneous nodules turning out to be a stage IVAE follicular T-cell lymphoma (aberrant co-expression of CD79a) with cutaneous and disseminated LN manifestation coupled with simultaneous EBV and CD30+ B cell proliferation. After 6x (R-)CHOEP, a CR was achieved; however, within 3 months, a very early relapse occurred. The patient was enrolled and randomized into the experimental arm (nivolumab, gemcitabine, and oxaliplatin) of the NIVEAU trial (29) but suffered from distinct PD after an initial mixed response. As a third-line treatment, MTX weekly was administered, however without any response. Finally, treatment was rotated to TEPIP, and the patient developed CR which lasted for 10 months. During this period, no relevant adverse events occurred. Upon progression, the patient underwent involved site radiation and belinostat treatment followed by short episodes of bendamustine and brentuximab-vedotin treatment, but no further response was achieved.

Conclusion: Patient case 3 demonstrates a long-lasting response without adverse events by TEPIP in a heavily pre-treated and refractory patient with follicular T-cell lymphoma in nodal and extranodal lesions.

#### Case 4 (pt. 11)

**Figure 4:**
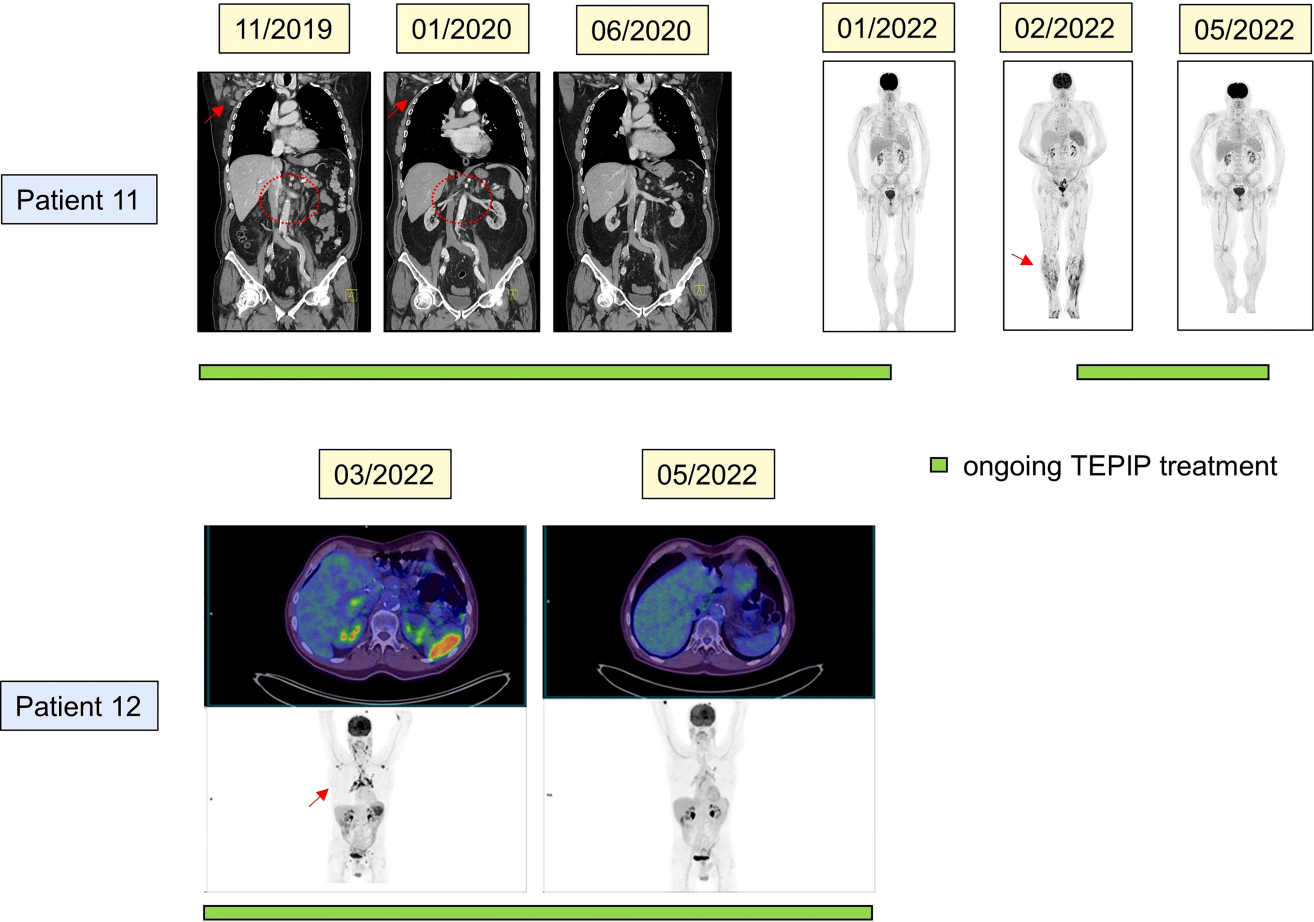
Clinical course of two exemplary patients (pt. 11: upper panels; pt. 12 lower panels) as assessed by radiological imaging. Depicted are the treatment responses at the indicated timepoints. Computed tomography and positron emission tomography were utilized. Green bars denote time on trofosfamide, etoposide, procarbazine, idarubicin, and prednisolone treatment. Red arrows and dotted circles indicate lymphoma manifestations.

A 66-year-old male patient with a history of prostatectomy due to prostate carcinoma and with a persistent single osteoblastic osseous metastasis (left os ilium) presented to our department with a first diagnosis of stage III AITL with exclusive lymph node manifestation. After five courses of CHOP, peripheral blood stem cell (PBSC) apheresis between each course of DHAP and DHAC was performed. At the timepoint of HD chemotherapy (BEAM) and ASCT, the patient was in complete remission. Within 6 months upon ASCT, early relapse occurred, and despite two courses of salvage treatment with gemcitabine and oxaliplatin, the disease progressed. The PSMA-PET result confirmed a stable disease (M1 oss.) of prostate carcinoma. Due to AITL progression, treatment was rotated to all-oral TEPIP (q4w, d1-7), and complete and durable remission (24 months) was achieved within two courses (**Figure 4**). Finally, treatment was terminated due to long-lasting CR after 2 years. Within the following 3 months, the patient relapsed with lymphoma infiltration of the skin, lymph nodes, and bone marrow (stage IV). At the same timepoint, a myelodysplastic neoplasm (MDS-RS-MLD) was incidentally diagnosed. TEPIP was re-initiated in five-weekly courses, resulting in a renewed very good PR within 3 months (**Figure 4**). Due to MDS-related cytopenia, TEPIP was replaced with 5-azacitidine. However, the patient died due to progression to AML at 5 months later.

Conclusion: Patient case 4 illustrates TEPIP as a safe and potent treatment option in AITL even after ASCT and with concomitant metastasized prostate carcinoma. In addition, it underpins its ability to reinduce remission after relapse.

#### Case 5 (pt. 12)

A 66-year-old pre-diseased (CVD, s/p esophagogastric junctional adenocarcinoma) male patient presented with stage IV A ALK-negative, CD30-positive ALCL with lymph node, cutaneous, and pulmonary manifestations, and treatment was initiated with BV-CHP. Between courses 2 and 3, PBSC apheresis was performed. After six courses, only PR was achieved, and within a further 2 months of watchful waiting, the disease distinctly progressed with new liver, spleen, and testicular manifestations. Due to a reduced general condition and his medical history, an intensive salvage or high-dose regimen was discarded, and low-dose TEPIP was administered (q4w, d1-7). After two courses, a PR was observed (**Figure 4**). However, the patient developed neurologic symptoms (*e*.*g*., amnesic aphasia) which later turned out to be a symptom of transient ischemic attack/stroke. After the interruption of treatment, the patient rapidly relapsed within 3 months and passed away.

Conclusion: Patient case 5 demonstrates the therapeutic option of TEPIP in ALCL when treatment with ALK inhibitors is not reasonable or possible.

### Safety and toxicity profile of TEPIP therapy

Adverse events emergent to TEPIP were observed in eight patients (4/8 CTCAE ≥grade 3) as documented in **Table 3**. Treatment-related deaths did not occur. One patient (pt. 11) died from concomitant secondary acute myeloid leukemia arising from myelodysplastic syndrome. The development of MDS/secondary acute myeloid leukemia was rated to be most likely attributed to multiple previous therapies including high-dose chemotherapy; nevertheless, a causal relation between MDS/AML and TEPIP is also possible. Grade 4 toxicity was only observed in one patient developing severe leukopenia, which improved after the discontinuation of TEPIP therapy. The most frequent toxicities overall were fatigue, thrombopenia, and elevated levels of liver transaminases, with the latter two additionally representing the most frequent grade 3 toxicities. In four patients, TEPIP was permanently ceased: the reasons were hepatotoxicity (pt. 5 and 6), cytopenia (pt. 5 and 6), and renal toxicity (pt. 7) coupled with insufficient responses to therapy, whereas one patient (pt. 12) developed neurological toxicity manifesting itself as amnesic aphasia. Nevertheless, this patient suffered from long-standing atrial fibrillation and pronounced atherosclerosis, which could have crucially contributed to the occurrence of neurological toxicity. Furthermore, dose reductions of trofosfamide owing to intolerable fatigue (pt. 3 and 11) or dysuria (pt. 2) were required in three patients, while dose reduction of etoposide and idarubicin due to fatigue was necessary in one patient (pt. 11). After the dose modifications, increase of treatment intervals, or discontinuation of TEPIP treatment, almost all toxicities appeared to be at least partially reversible. One patient (pt. 3) developed cutaneous squamous cell carcinoma (cSCC) lesions on both arms, prompting the cessation of TEPIP therapy to boost wound healing after the surgical removal of cSCC. In summary, TEPIP treatment is associated with a tolerable safety profile qualifying for use in an outpatient setting.

**Table 3:** Treatment-emergent adverse events.

Patient ID	Toxicity	Grade^a^	Response	Outcome
1	Fatigue	1	Temporary discontinuation of TEPIP	Improvement
2	Thrombopenia	2	Temporary discontinuation of TEPIP	Improvement
	Dysuria	2	Dose reduction of trofosfamide and prednisolone	Improvement
3	Fatigue	2	Dose reduction of trofosfamide and longer treatment intervals of 6 weeks	Improvement
	Cutaneous squamous cell carcinoma	Not applicable	Discontinuation of TEPIP	Improvement after surgical excision
4	None	–	–	–
5	Elevated liver transaminases	3	Discontinuation of TEPIP	Resolution
	Leukopenia	4	Discontinuation of TEPIP	Improvement
	Anemia	3	Discontinuation of TEPIP	Improvement
	Thrombopenia	3	Discontinuation of TEPIP	Improvement
6	Elevated liver transaminases	3	Discontinuation of TEPIP	Improvement
	Thrombopenia	3	Discontinuation of TEPIP	Persistence
7	Infection (urinary)	3	Discontinuation of TEPIP	Improvement
	Elevated creatinine	3	Discontinuation of TEPIP	Improvement
8	None	–	–	–
9	None	–	–	–
10	None	–	–	–
11	Fatigue MDS-RS-MLD	2 Not applicable	Discontinuation of Idarubicin and dose reduction of trofosfamide and etoposide -	Improvement Progression to AML
12	Neurological symptoms (word finding disorder) originating from stroke	3	Discontinuation of TEPIP	Improvement

a According to Common Terminology Criteria of Adverse Events Version 5 (2017).

## Discussion

Treatment of T-cell lymphoma in elderly and frail patients poses a challenge to clinicians. While first-line treatment with age-adjusted, dose-attenuated multi-agent chemotherapy (*e*.*g*., miniCHOP) yields disappointing therapeutic success with only low PFS and OS rates (13), the next-line options are limited as the respective patients are not eligible for intensive salvage treatment, HD chemotherapy, or stem cell transplantation. As a result, there is medical need for palliative regimens considering the necessity of effective low-dose approaches while meeting many patients’ request for outpatient concepts.

In the study presented here, we show data from an elderly (median, 70 years) cohort of 12 patients with PTCL being under treatment with an all-oral, low-dose chemotherapy regimen TEPIP at the Department of Hematology of the University Medical Center Regensburg over the past decade. TEPIP comprises four oral chemotherapeutic drugs (trofosfamide, etoposide, procarbazine, and idarubicin) plus steroids (prednisolone or dexamethasone), each of which has been proven effective in the treatment of non-Hodgkin lymphoma disease (7, 27, 30–33). The majority of the cohort is characterized by poor prognosis with high-intermediate or high IPI scores and extensive disease (Ann Arbor stage III or IV) at TEPIP onset. Treatment with the TEPIP regimen followed a median of 1.5 prior treatment lines (range, 0–4), and a median of 2.5 TEPIP courses was applied. An ORR of 42% was achieved; however, an equally large group of patients experienced primary treatment failure. Overall survival reached a median of 6.2 months (185 days) with one patient still being alive in sustained complete remission. Anecdotal observations of responding patients (as demonstrated in the brief patient cases) highlight the potential benefit of TEPIP treatment in complex everyday settings, such as frail patients with relapse, refractory disease in various PTCL subtypes, and patients with a concurrent solid malignancy. Remarkably, several patient cases have shown rapid relapse after the interruption or discontinuation of therapy, but partial remission was achieved at least in one case by resuming TEPIP therapy.

All but one patient received TEPIP at a relapsed or refractory stage, which historically has a dismal prognosis. Mak et al. reported a median OS in R/R PTCL of 5.5 months (11), which was still confirmed 5 years later and more favorable only in a selected patient cohort with unimpaired performance status who were receiving salvage chemotherapy (12). This is particularly important for elderly patients who are not eligible for intensive first-line (and next-line) treatment due to their increased susceptibility to chemo-associated toxicities and, as a result, are more often likely to have primary refractory or relapsed diseases as reflected by the reported 2-year PFS of 37% (13). FDA-approved single-agent R/R treatment strategies like anti-folate pralatrexate or HDAC inhibitors romidepsin and belinostat have shown ORR of 25% to 29% and DOR of 10 to 17 months (15–17), not exceeding the ORR observed with TEPIP treatment. Better ORR could be achieved with the antibody–toxin conjugate brentuximab–vedotin (BV). However, studies required CD30 positivity or an ALCL phenotype (34, 35), which, in turn, limits the availability to a selected patient population. Additionally, most novel drugs are administered intravenously and require close monitoring of side effects or through in-patient treatment. Furthermore, promising results demonstrating the efficacy of hypomethylating agents (HMA) in PTCL with mutations in epigenetic regulators (*e*.*g*., DNTM3A, TET2, and IDH2) have been obtained in smaller studies (18, 36), raising hope for precise, molecularly tailored treatment regimens. In this context, oral HMA preparations, in particular, represent a promising approach for future outpatient strategies and are the subject of current trials (NCT04747236 and NCT03161223). However, robust data are still pending, and the potential role in a therapeutic sequence remains unclear. As of now, neither HDAC inhibitors nor HMA has received approval from the EMA for use in T-cell lymphoma. Thus, the second-line treatment of elderly/frail patients with R/R PTCL requires a broad therapeutic repertoire to enable individual treatment. Cox and colleagues have recently shown that the all-oral chemotherapy regimen DEVEC (prednisolone, etoposide, vinorelbine, and cyclophosphamide) achieves impressive ORR/CR (66%/25%) and OS (13 months) with a tolerable rate of adverse events in an elderly cohort of R/R PTCL with poor prognosis (IPI ≥3: 75%) (24). The outperformance in terms of ORR and OS despite a comparable cohort might be at least partially explained by a higher median number of therapy courses in the DEVEC (median, 8.5 courses) compared with our study (median, 2.5 courses), underscoring the highly palliative intention to treat in the TEPIP cohort. Furthermore, DEVEC was administered in a metronomic manner, potentially leading to differential, pleiotropic pharmacodynamics (37, 38). In recognition of the outstanding DEVEC results, a metronomic application of TEPIP with lower doses, more regular administration, and reduced drug-free brakes (39, 40) should be evaluated in future studies. There are few other reports of low-dose, all-oral treatment regimens in PTCL (25–27), emphasizing the need for more outpatient options in advanced palliative settings that provide a decentralized treatment approach, particularly for rural areas. Importantly, oral HMA and TEPIP may even pose an appropriate palliative first-line therapy for PTCL in elderly/frail patients in case of rejection of intravenous chemotherapy, which requires regular intravenous access and presentation to specialized oncological facilities. For patients declining systemic therapy in favor of best supportive care, all-oral therapy with TEPIP might be discussed in the first line.

In our study, treatment-emergent adverse events (TE-AE) CTCAE ≥ grade 3 were observed in 33% of patients, with one grade 4 (leukopenia) but no fatal TE-AE. Relevant infectious disease only occurred in one patient, which is most likely due to the recommended dose adjustments in case of cytopenia. As this was performed according to the physician’s choice, reliable numeric statements unfortunately are not available. One patient with long-term TEPIP treatment (pt. 3) developed multiple cSCC, which was classified as possibly related to treatment. However, a retrospective analysis of the patient’s previous circumstances (*e*.*g*., sun exposure) was not possible. Another patient (pt. 11) died from MDS/AML 5 months after re-discontinuation of TEPIP, which occurred concurrently with PTCL relapse after an initial complete response and prolonged 2 years of TEPIP treatment (**Figure 1**). TEPIP includes alkylating agents (procarbazine and trofosfamid) and topoisomerase II inhibitors (etoposide and idarubicin), which are known to cause secondary MDS and AML in a group of patients after the treatment of hematologic malignancies (41, 42). Thus, we recognize a potentially therapy-related event. However, one must consider the four prior treatment regimens, including high-dose BEAM, in this patient. Furthermore, in the recently reported cohort of patients with DLBCL treated with TEPIP, no patient suffered from secondary malignancies (1). Hypothetically, an additional potentially contributing factor to the occurrence of MDS and AML in the cohort of patients with PTCL treated with TEPIP might derive from potential clones with pre-lymphomatous TET2 and DNMT3A mutations. Those genetical alterations are frequently present in patients diagnosed with AITL, and AITL *per se* is associated with a certain co-occurrence of myeloid neoplasias (43). Nonetheless, we recommend that the maintenance of therapy be regularly and carefully reviewed. Notably, the same patient started TEPIP while suffering from an osseous metastasized prostate carcinoma which has not been progressing despite a long-term treatment. In summary, compared with previous palliative treatment regimens (15–17, 34), the safety profile of TEPIP is tolerable and manageable. Due to retrospectivity and the outpatient setting, additional unreported events cannot be completely excluded.

The rapid relapse after discontinuation in patient 4 with a subsequent re-induction of remission stirs the question for TEPIP maintenance therapy. A limiting factor for continuous therapy is set by the cumulative dose of idarubicin to prevent anthracycline-associated cardiomyopathy. Thereafter, TEPP without idarubicin could pose a backbone for long-lasting maintenance. In clinical practice, we advocate the continuation of TEPIP/TEPP therapy past remission induction until disease progression or the occurrence of unacceptable toxicity.

We are aware of the limitations of the study derived from the small, heterogenous cohort, a partially incomplete data set due to the palliative outpatient setting, retrospectivity without a control group as well as the short median duration of TEPIP treatment, which negatively affect the explanatory power of our results. R/R PTCL is a rare disease, and the clinical courses of the reported cohort span more than a decade (2010–2022), partially explaining the incomplete histopathologic reports (examination of molecular profiles or antigen expression) that may impact treatment decisions today. Additionally, the currently available treatment guidelines were published after the first patients were treated. All patients included in this study were treated individually and independently on a compassionate-use basis. Hence, individual treatment regimens differ, compromising the direct comparability of patients as well as the generation of pooled analyses for common outcome parameters, such as overall survival and progression-free survival. Moreover, no fixed and pre-established parameters for the evaluation of safety and efficacy are present. Thus, the direct comparability of patients is compromised.

In conclusion, the TEPIP regimen presented is a treatment option for patients with R/R PTCL that is both effective and well tolerated, with a focus on the quality of life in advanced palliative settings. We encourage the initiation of controlled clinical trials to prospectively evaluate TEPIP and gain further experience.

## Data availability statement

The raw data supporting the conclusions of this article will be made available by the authors without undue reservation.

## Ethics statement

The analysis was approved by the University of Regensburg (reference number: 23-3250-104) and performed in compliance with the current Declaration of Helsinki. All cases analyzed were pseudonymized, and patients who were alive gave written informed consent for publication.

## Author contributions

MF, DCH, BZ, FL, SM, WH, MV, AR and DH treated the patients. MF, DCH and DH analyzed the data and wrote the manuscript. All authors contributed to the article and approved the submitted version.
